# Ambient LED Light Noise Reduction Using Adaptive Differential Equalization in Li-Fi Wireless Link

**DOI:** 10.3390/s21041060

**Published:** 2021-02-04

**Authors:** Yong-Yuk Won, Sang Min Yoon, Dongsun Seo

**Affiliations:** 1 Department of Electronic Engineering, Myongji University, 116 Myongji-ro, Cheoin-gu, Yongin 17058, Korea; sdsphoto@mju.ac.kr; 2 College of Computer Science, Kookmin University, 77 Jeongneung-ro, Seongbuk-gu, Seoul 02707, Korea; smyoon@kookmin.ac.kr

**Keywords:** adaptive differential equalization, ambient light noise, digital signal processing, light-emitting diodes, Li-Fi, visible light communication

## Abstract

For Li-Fi wireless links based on a white light emitting diode, an adaptive differential equalization (ADE) technique that reduces various noises such as interference noise and shot one generated from ambient light sources is pro-posed. The ADE technique reduces noise by taking advantage of the fact that the derivative between adjacent sampling points of signal with digital waveform is very different from that of noise with the random analog waveform. Furthermore, a weighting function that reflects the Poisson characteristics of shot noise is applied to the ADE technique in order to maximize the reduction efficiency of ambient noise. The signal-to-noise ratio of input non-return-to-zero-on–off keying (NRZ-OOK) signal is improved by 7.5 dB at the first-generation forward error correction (FEC) threshold (the bit error rate (BER) of 8 × 10^−5^) using the optical wireless experimental link. In addition, it is confirmed that it is possible to maintain the transmission performance corresponding to the BER of 1 × 10^−5^ by using the proposed ADE technique, even when the intensity of the ambient light source increases by 6 dB.

## 1. Introduction

The multimedia services of the 5G network have begun since 2019, and a 6G network, which is expected to increase 100 times more than the transmission rate of the 5G network, will be implemented soon [1,2,3]. It is easy to predict that the transmission capacity of the wired network will increase in line with the establishment of the 6G wireless network [4,5,6]. In such a wired/wireless network environment, a massive Internet of things (IoT) service in which a large number of mobile devices and smart sensors are connected is being partially operated and is evolving further [7,8,9]. It is important that these massive IoT services are implemented in various environments in order to spread to various fields and stabilize quickly. In most environments, it is possible to implement mobile services using RF(radio frequency) signals. However, it is necessary to use other types of signals in spaces where RF signals are not sufficiently transmitted or difficult to use. In particular, it may be difficult to use an RF signal-based wireless service in both an industrial facility where high security is required and a medical facility where there is no electromagnetic interference. In addition, since it is expected that RF frequency resources with a frequency band of up to 300 GHz will be gradually depleted in the future, it is expected that auxiliary services through other transmission media will be needed in order to fully support these IoT services. Among these auxiliary technologies, a light fidelity (Li-Fi) transmission technology, which transmits data wirelessly using light emitted from a white light-emitting diode (LED), is capable of operating IoT services within a special environment such as a hospital and national security facilities [10,11]. In the past 20 years, Li-Fi related research has shown great growth and potential [12]. Until now, the Li-Fi transmission system has generally shown a transmission rate of about 1 Gb/s at a wireless transmission length of 10 m in a building environment [13,14,15,16]. In general, the transmission rate is determined by the signal-to-noise ratio (SNR) of the received signal. Furthermore, the important thing is that the SNR is determined according to the ambient noise [17]. In the case of Li-Fi, the cause of reducing transmission performance is known as noise generated by ambient light sources [18,19,20,21]. There are two types of noise generated from ambient light sources. The first is a shot noise generated by a random change in the number of photons incident on the optical receiver. The second is interference noise that changes with time generated by an electronic circuit driving a light source [19,20]. The impact of noise from the above-mentioned ambient light sources on Li-Fi transmission performance has been reported in [18,20].

Moreira et al. have studied the impact of interference noise generated from fluorescent and incandescent lamps on the transmission performance of optical signals in infrared wireless links. However, the effect of contemporary white light sources such as white LEDs, dimmable LEDs, and energy-saving bulbs on the visible light communication channel is not covered [18]. A.C. Boucouvalas analyzed the RF spectrum of interference noise generated from various artificial light sources in indoor wireless optical links based on the IR range, including tungsten bulbs, IR headphones, TV remotes, and non-visual daylight [22]. M. Galal analyzed the shot and interference noise generated from white LEDs, dimmable LEDs, and energy-saving light bulbs, which are recently used as lighting, and implemented relevant modeling [20]. However, a few techniques have been proposed to reduce shot and interference noise from these ambient light sources [23,24,25]. Adiono designed and implemented a high-frequency filter based on a DC offset remover circuit to reduce interference noise from ambient light sources [23]. However, no data transmission experiment was performed, and only the noise reduction was experimentally verified. In addition, only incandescent and fluorescent lamps were employed as ambient light sources, and no other LED light sources were used. In [24], an algorithm, which optimizes the SNR in the visible light communication system, was implemented assuming a multipass channel as an optical wireless channel. It was verified through simulation. Unfortunately, the noise generated by the ambient light source was not taken into account. Pham proposed a technique to reduce the noise generated by the ambient light source by implementing the average voltage tracking technique as a circuit [25]. However, only fluorescent lamps were used as an ambient light source except for other LED lamps. The average voltage tracking method was verified using an NRZ-OOK signal of 1 Mb/s.

In this paper, we propose a technique to increase the SNR of a received signal by reducing the noises generated from the ambient light sources that are simultaneously introduced during optical wireless transmission in a Li-Fi link based on white LED. As two ambient light sources, a fluorescent lamp and dimmable LED bulb light are used. A non-return to zero-on off keying (NRZ-OOK) signal is used as data for the optical wireless transmission experiment on the Li-Fi link. The noises from the above-mentioned light sources used in the experiment are shot noise with Poisson probability function and interference noise in the form of a composite sinusoidal function that changes with time.

The proposed technique attenuates the magnitude of the noise by using the fact that the derivative function of the user data with digital waveform is very different from that of the noise with analog waveform. Furthermore, they change each other differently with time. In other words, in the case of a digital signal, its derivative value is very small in a range having an intensity of 1 or 0, whereas, in the case of an analog signal, its derivative value varies randomly. Moreover, the proposed technique increases the efficiency of reducing shot noise by using the Poisson function as a weight function. The proposed technique does not use matched RF filters and is implemented based on digital signal processing, so the intensity of the signal does not decrease due to insertion loss. In addition, since it is not necessary to know the header information of the input signal in advance to reduce the noise of the ambient light source, it can be applied to networks having various protocols. We implement the experimental optical wireless link for Li-Fi in order to verify the proposed technique. The SNR, bit error rate (BER), and eye diagram of the NRZ-OOK signal are measured to find if the noises from the surrounding light sources are reduced using the proposed technique. Here, we call the proposed technique as adaptive differential equalization (ADE).

## 2. Ambient Light Noise Model and Adaptive Differential Equalization

It is important to implement mathematical modeling of both the ambient light noise and the proposed technique to theoretically verify that the proposed technique compensates for the deterioration of the transmission performance caused by the interference noise generated by the ambient light source. Section 2.1 describes the time-varying equations of the interference noise generated by the two light sources (fluorescent lamp and dimmable LED) mentioned in the introduction, and the proposed technique explains the noise-reduction process mathematically in Section 2.2.

### 2.1. Interference and Shot Noise Model of Fluorescent Lamp and Dimmable LED

**Figure 1:**
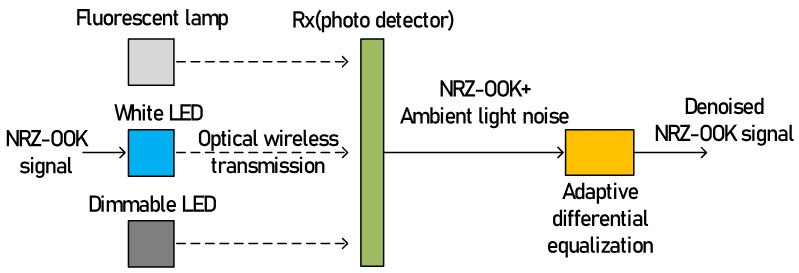
Ambient light noise reduction using adaptive differential equalization.

Figure 1 shows the process of reducing interference noise generated from ambient light sources (fluorescent lamp and dimmable LED) using the proposed adaptive differential equalization technique in the Li-Fi wireless link. The white LED light exhibits similar characteristics to an uncorrelated broadband optical source, such as amplified spontaneous emission (ASE). Therefore, the white LED light, $E_{s}\left(t\right)$ directly modulated by the NRZ-OOK signal and two ambient lights (fluorescent lamp, $E_{flu}\left(t\right)$ and dimmable LED, $E_{dim}\left(t\right)$) with interference, noises can be expressed as Equations (1)–(3), respectively [26].

(1) $$E_{s}\left(t\right)=A\left(t\right)\sum_{k=-M_{0}}^{M_{0}}exp\left[j\left\{2\pi \left(f_{0}+k\delta f_{0}\right)t+\phi_{k}\right\}\right]$$

(2) $$E_{flu}\left(t\right)=N_{1}\left(t\right)\sum_{k=-M_{1}}^{M_{1}}exp\left[j\left\{2\pi \left(f_{1}+k\delta f_{1}\right)t+\theta_{k}\right\}\right]$$

(3) $$E_{dim}\left(t\right)=N_{2}\left(t\right)\sum_{k=-M_{2}}^{M_{2}}exp\left[j\left\{2\pi \left(f_{2}+k\delta f_{2}\right)t+\psi_{k}\right\}\right]$$

where A(t) is an NRZ-OOK signal, $N_{1}\left(t\right)$ and $N_{2}\left(t\right)$ are interference noises, which are generated from fluorescent lamps with electrical circuits and dimmable LEDs, including LED driver circuits with a dimming controller. $f_{0}$, $f_{1}$, and $f_{2}$ are the center optical frequencies in the spectral region of three different lights. $\delta f_{0}$, $\delta f_{1}$, and $\delta f_{2}$ are elemental frequency intervals of each light. $\phi_{k}$, $\theta_{k}$, and $\psi_{k}$ are random optical phases uncorrelated for each k. $B_{0}$, $B_{1}$, and $B_{2}$ are the spectral regions of each light. $M_{0}$ is $B_{0}/2\delta f_{0}$. $M_{1}$ is $B_{1}/2\delta f_{1}$, and $M_{2}$ is $B_{2}/2\delta f_{2}$. Accordingly, the received optical signal, $E_{r}\left(t\right)$ in the receiver (Rx) can be described as Equation (4).

(4) $$E_{r}\left(t\right)=E_{s}\left(t\right)+E_{flu}\left(t\right)+E_{dim}\left(t\right)$$

During the optical to electrical conversion in the photodetector of Rx, shot noise is generated in addition to the NRZ-OOK signal and interference noise. Here, two different types of shot noise are generated in Rx. In more detail, they are stationary shot noises generated in the white LED as well as two ambient lights biased by DC current and non-stationary shot noises from the time-varying photocurrent generated by the NRZ-OOK signal and interference signal. The time-varying and non-stationary shot noise current can be realized by using Equation (5) by Personic analysis [27]:

(5) $$I_{shot}\left(t\right)=\frac{ℛ\int_{-\infty }^{\infty }p\left(\tau \right)h_{I}^{2}\left(t-\tau \right)d\tau }{2B}$$

where ℛ is the responsivity of the photodetector, q is the electronic charge, p(t) is the received optical power and $h_{I}\left(t\right)$ is the impulse response of the optical receiver, including bias circuit, transimpedance amplifier and equalizer.

The output photocurrent, including interference noise and two kinds of shot noise, can be expressed as Equation (6) [28]:

(6) $$\begin{array}{c} I_{r}\left(t\right)=ℛ{\left\{\frac{E_{s}\left(t\right)+E_{flur}\left(t\right)+E_{dim}\left(t\right)}{\sqrt{2}}\right\}}^{2}\\ =\frac{ℛ}{2}\left\{\begin{array}{c} A\left(t\right)+N_{1}\left(t\right)+N_{2}\left(t\right)+\frac{ℛ\int_{-\infty }^{\infty }E_{s}^{2}\left(\tau \right)h_{I}^{2}\left(t-\tau \right)d\tau }{4B}+\\ \frac{ℛ\int_{-\infty }^{\infty }E_{flur}^{2}\left(\tau \right)h_{I}^{2}\left(t-\tau \right)d\tau }{4B}+\frac{ℛ\int_{-\infty }^{\infty }E_{dim}^{2}\left(\tau \right)h_{I}^{2}\left(t-\tau \right)d\tau }{4B}+I_{dc-shot} \end{array}\right\} \end{array}$$

where $I_{dc-shot}$ is a non-varying stationary shot noise caused by a photodetector, B is 3 dB bandwidth of photodetector.

The terms from the fourth to the sixth on the right side of Equation (6) refer to the non-stationary shot noise generated by the photocurrent that changes due to the NRZ-OOK signal and interference noise, respectively. For reference, it was reported that a non-stationary shot noise generated by a time-varying photocurrent from a modulated light has a white spectrum with a Poisson probability distribution and has a correlation with a modulated signal [29]. Galal and Moreira discovered that the second and third terms on the right-hand side of Equation (6), the interference noise generated by other types of LED lights, can be expressed using the Fourier series as Equations (7) and (8) [19,20]:

(7) $$N_{1}\left(t\right)=\sqrt{2\left[a_{0}+\overset{n1}{\underset{i=1}{\sum }}\begin{array}{c} a_{i}\cos\left(2\pi \left(100i-50\right)t\right)\\ +b_{i}cos\left(2\pi \left(100i\right)t\right) \end{array}\right]}$$

(8) $$N_{2}\left(t\right)=\sqrt{2\left[c_{0}+\overset{n2}{\underset{i=1}{\sum }}\begin{array}{c} c_{i}\cos\left(2\pi \left(100i-50\right)t\right)\\ +e_{i}cos\left(2\pi \left(100i\right)t\right) \end{array}\right]}$$

where $a_{0}$ and $c_{0}$ are the average DC value, $a_{i}$, $b_{i}$, $c_{i}$, and $e_{i}$ are the amplitudes of the 50 Hz and 100 Hz harmonics, respectively. n1 and n2 are the numbers of the harmonics that can affect the SNR of the data signal.

Here $a_{0}$ and $c_{0}$ can be simply removed using a DC block as an RF device. However, it is not possible to remove $a_{i}$ and $b_{i}$ using a high-pass filter because the 50 Hz and 100 Hz harmonic distortions corresponding to $a_{i}$ and $b_{i}$ overlap the bandwidth of the NRZ-OOK signal. In addition, since shot noise generated by NRZ-OOK and interference noise also is generated over the entire band, it plays a role in reducing the signal-to-noise ratio of the NRZ-OOK signal. Hence, we propose the ADE technique to reduce the interference noise showing as harmonic distortion as well as two kinds of shot noise.

As the distance between two light sources (fluorescent lamp and dimmable LED) generating interference noise and Rx increases, the intensity of interference noise increases because the optical power injected into Rx increases. On the other hand, as the distance between the white LED modulated by the NRZ-OOK signal and Rx gets closer the optical signal-to-noise ratio increases. In general, the optical power received in an optical wireless transmission is inversely proportional to the square of the distance. Therefore, it is desirable to implement a wireless environment where the distance between the white LED and Rx is small, and the distance to other light sources is increased if possible.

### 2.2. Interference and Shot Noise Reduction Using Adaptive Differential Equalization

In general, when an arbitrary signal (*x*) is transmitted through any transmission medium, noise (*d*) generated during the transmission is added, and then the output signal (*y*) is produced as described in Equation (9).

(9) y=x+d

where y is an output signal, x is an input signal, d represents the noise generated in the process of passing through the transmission medium. Using Equations (6)–(8), Equation (9) can be summarized as Equation (10):

(10) $$\begin{array}{c} \underset{y}{\underset{︸}{I_{r}\left(t\right)}}=\underset{x}{\underset{︸}{\frac{ℛ}{2}A\left(t\right)}}+\underset{d}{\underset{︸}{\frac{ℛ}{2}\sqrt{2\left[a_{0}+\sum_{i=1}^{n1}\begin{array}{c} a_{i}\cos\left(2\pi \left(100i-50\right)t\right)\\ +b_{i}\cos\left(2\pi \left(100i\right)t\right) \end{array}\right]}}}+\\ \underset{d}{\underset{︸}{\frac{ℛ}{2}\sqrt{2\left[c_{0}+\sum_{i=1}^{n2}\begin{array}{c} c_{i}\cos\left(2\pi \left(100i-50\right)t\right)\\ +e_{i}\cos\left(2\pi \left(100i\right)t\right) \end{array}\right]}+\frac{{ℛ}^{2}\int_{-\infty }^{\infty }E_{s}^{2}\left(\tau \right)h_{I}^{2}\left(t-\tau \right)d\tau }{8B}}}+\underset{d}{\underset{︸}{\frac{{ℛ}^{2}\int_{-\infty }^{\infty }E_{flur}^{2}\left(\tau \right)h_{I}^{2}\left(t-\tau \right)d\tau }{8B}}}+\\ \underset{d}{\underset{︸}{\frac{{ℛ}^{2}\int_{-\infty }^{\infty }E_{dim}^{2}\left(\tau \right)h_{I}^{2}\left(t-\tau \right)d\tau }{8B}+\frac{ℛ}{2}I_{dc-shot}}} \end{array}$$

As shown in Equation (10), an NRZ-OOK signal with a digital waveform is designated as an input signal (*x*), and the interference noise and shot noise (*d*) generated during the optical wireless transmission are expressed as harmonic distortion and random white noise with Poisson function, respectively.

In this way, various signal processing techniques based on total variation (TV) techniques have been proposed to reduce the corresponding noise when the waveform of the input signal is different from that of the noise [30,31,32]. If a conventional TV technique is used to recover the NRZ-OOK signal, Equation (10) is changed into Equation (11):

(11) $$\begin{array}{c} \hat{A\left(t\right)}=\underset{P\left(t\right)}{\mathrm{argmin}}\|\frac{ℛ}{2}A\left(t\right)-I_{r}\left(t\right){\|}_{2}^{2}+\lambda {\left\|A(t)\right\|}_{TV}\\ =\underset{P\left(t\right)}{\mathrm{argmin}}\|\frac{ℛ}{2}\sqrt{2\left[a_{0}+\sum_{i=1}^{n1}\begin{array}{c} a_{i}\cos\left(2\pi \left(100i-50\right)t\right)\\ +b_{i}\cos\left(2\pi \left(100i\right)t\right) \end{array}\right]}\\ \;\;\;\;+\frac{ℛ}{2}\sqrt{2\left[c_{0}+\sum_{i=1}^{n2}\begin{array}{c} c_{i}\cos\left(2\pi \left(100i-50\right)t\right)\\ +e_{i}\cos\left(2\pi \left(100i\right)t\right) \end{array}\right]}\\ \;\;\;\;\;+\frac{{ℛ}^{2}\int_{-\infty }^{\infty }E_{s}^{2}\left(\tau \right)h_{I}^{2}\left(t-\tau \right)d\tau }{8B}+\frac{{ℛ}^{2}\int_{-\infty }^{\infty }E_{flur}^{2}\left(\tau \right)h_{I}^{2}\left(t-\tau \right)d\tau }{8B}\\ \;\;\;\;\;\;\;\;\;+\frac{{ℛ}^{2}\int_{-\infty }^{\infty }E_{dim}^{2}\left(\tau \right)h_{I}^{2}\left(t-\tau \right)d\tau }{8B}+\frac{ℛ}{2}I_{dc-shot}{\|}_{2}^{2}+\lambda {\left\|A\left(t\right)\right\|}_{TV} \end{array}$$

where ${\left\|\;\right\|}_{TV}$ is TV norm. Thus, ${\left\|A\left(t\right)\right\|}_{TV}=\int_{\mathbb{R}}^{}\left|\nabla A\left(t\right)\right|dt$ with the gradient operator ∇. The regularization parameter, λ is used to optimize the relationship between the recovered NRZ-OOK signal waveform and SNR.

An NRZ-OOK signal, $\hat{A\left(t\right)}$ is recovered when the first term on the right side (interference noise and shot noise) of Equation (11) is minimized. However, since the conventional TV technique such as Equation (11) clearly reduces the noise that has a variance of the white Gaussian function, it is not possible to effectively reduce harmonic distortions showing a complex sinusoidal function and shot noise with a Poisson distribution.

To solve this issue, we proposed the ADE technique that applies a higher-order approximation function to the first term on the right side of Equation (11). The ADE technique is implemented by using the least-squares method that is less dependent on the regularization parameter and adapts to the waveform of the signal, and a non-local weighting function that is robust to noise with a random analog waveform. In addition, the Poisson distribution function was added to the weighting function to reduce the non-stationary and stationary shot noise effectively.

The ADE technique is mathematically formulated as follows: Since it is implemented based on digital signal processing (DSP), the NRZ-OOK signal, interference noise, and shot noise are digitally sampled. Therefore, when converting from continuous-time signal to discrete-time signal, the restored NRZ-OOK signal, $\hat{A\left(t\right)}$, is changed to $\hat{A\left(i\right)}$ in case that Ψ(i) is the whole set of sampling points, i. Here, the polynomial function, $\Gamma_{p}\left(i\right)$ mathematically implemented to recover the NRZ-OOK signal using the ADE technique can be expressed by Equation (12).

(12) $$\Gamma_{p}\left(i\right)=\underset{f\in {Χ}_{L}}{\mathrm{argmin}}\sum_{j\in \Psi \left(i\right)}{\left|f_{r}\left(i\right)-A\left(j\right)\right|}^{2}Τ\left(j,i\right)+\lambda {\left\|f\right\|}_{TV}$$

where ${Χ}_{L}$ is the set of all polynomial functions of degree L or less. Τ(j,i) is a weight function to which a Poisson distribution function is applied, as shown in Equation (13).

(13) $$Τ\left(j,i\right)=exp\left(-\frac{\sum_{\nu \in K}\|I_{r}\left(j+\nu \right)-I_{r}\left(i+\nu \right){\|}^{2}S_{\sigma }\left(\|\nu \|\right)}{h^{2}}\right)$$

where $S_{\sigma }$ is the Poisson distribution function with a standard deviation of σ. K is a small set of sampling points located around sampling points of i and j, and h is a filtering parameter. As a result, $\Gamma_{p}\left(i\right)$ obtained by using Equation (12) becomes a recovered NRZ-OOK signal.

The algorithm to implement the proposed ADE technique performs iterative computation until the difference between adjacent sampling points of digitally sampled NRZ-OOK signal becomes smaller than a preset threshold. In general, iterative operations increase the complexity of DSP and the execution time of the algorithm. Hence, we reduced the execution time by replacing the iterative computation process with the split Bregman adaptive least-squares method [33]. In addition, under the assumption that the amount of calculation can be increased, the proposed ADE technique recovers the waveform of the received NRZ-OOK signal to the closest match to that of the input signal by reducing the difference between adjacent sampling values. Furthermore, a weighting function reflecting the Poisson distribution function is applied to the proposed technique in order to reduce the shot noise effectively.

In summary, the proposed ADE technique is based on a conventional TV technique. The proposed ADE technique is based on a conventional TV technique. However, the conventional TV technique has the disadvantage that it is not sensitive to minute changes in the waveform because it is too dependent on the regularization parameter that controls the shape of the waveform. Furthermore, it is effective only for white Gaussian noise. Therefore, the proposed ADE technique applies a higher-order approximation function to the first term on the right side of Equation (11) derived based on the conventional TV technique, as shown in Equation (12). A Poisson weight function was also added to effectively reduce shot noise. In other words, we derived Equation (11) using a conventional TV technique and then derived Equation (12), a mathematical expression of the proposed ADE technique, in order to clearly show the process in which the proposed technique is implemented mathematically.

Even in a wireless transmission system exhibiting a transmission rate of more than several hundred Mb/s, the effect of interference noise may still be valid. In general, baseband signals generated by digital modulation such as NRZ-OOK and pulse amplitude modulation (PAM) have their main information concentrated in the low-frequency band near DC. Therefore, since most of the noise generated by the ambient light source, including shot noise, shows low-frequency noise characteristics, baseband signals are affected by interference noise regardless of the transmission rate.

The proposed technique makes it possible to reduce shot noise regardless of the transmission rate. This is due to the following reasons. The proposed scheme is implemented using Equations (12) and (13), and there are no terms related to the transmission rate in these equations. The proposed technique works only on the waveform of a signal based on digital sampling. In other words, it can be seen that the proposed scheme operates regardless of the transmission rate.

Furthermore, in Equation (13), we focused on reducing shot noise by adding the Poisson function, which is a probabilistic function of shot noise. Therefore, it is possible to reduce shot noise by using the proposed technique even at a higher transmission rate.

Fortunately, in the case of a high transmission rate, the bandwidth of the baseband signal increases due to the increase in the transmission rate, but the bandwidth of the interference noise becomes constant regardless of the bandwidth of the baseband signal. Therefore, the impact of interference noise on the baseband signal can be reduced.

However, according to the energy conservation law, its SNR decreases as the bandwidth of the base signal increases. This causes the influence of interference noise to increase. Therefore, in order to meet the SNR required by the system, it is necessary to increase the intensity of the baseband signal when the light from the white LED is directly modulated by this. However, if the modulation depth is increased by 1 or more, distortion may occur, and then the SNR of the baseband signal may be reduced.

As another method, the link budget of the optical wireless transmission system can be increased by increasing the output optical power of the white LED light. However, the intensity of the ambient light source also should be taken into account depending on the lighting environment in the building; as the intensity of the ambient light source increases, the intensity of interference noise increases.

Accordingly, in a system having a high transmission rate, it is necessary to configure its link in consideration of the reduction of the SNR according to the increase in bandwidth and the intensity of interference noise generated from ambient light at the same time.

## 3. Experimental Setup

**Figure 2:**
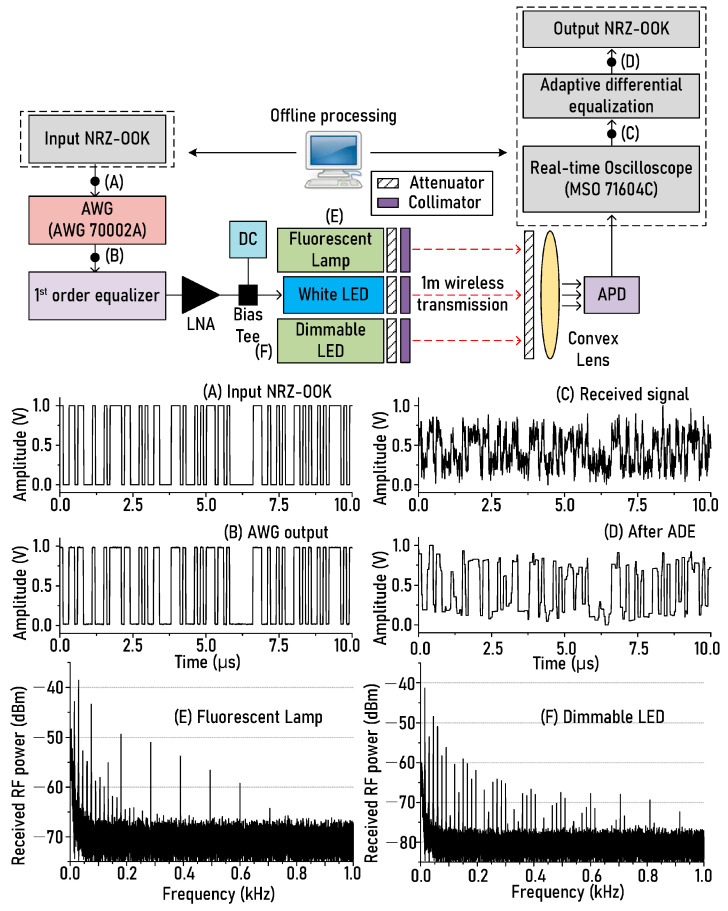
Optical wireless transmission link based on white light−emitting diode (LED) for the verification of adaptive differential equalization (ADE) technique. Insets below experimental setup: (**A**) input non−return−to−zero−on−off keying (NRZ-OOK) waveform, (**B**) output NRZ−OOK waveform from arbitrary waveform generator (AWG), (**C**) NRZ−OOK waveform received at avalanche photodiode (APD), (**D**) NRZ−OOK waveform after using the proposed ADE technique, (**E**) RF(radio frequency) spectrum of fluorescent lamp, (**F**) RF spectrum of dimmable LED.

Figure 2 shows the white LED-based optical wireless transmission link, which was implemented to experimentally verify the proposed ADE technique. The NRZ-OOK signal and the ADE technique were implemented using MATLAB^®^ (the part in the dashed box). The NRZ-OOK signal was generated using a pseudo-random bit sequence (PRBS) signal with a length of 2^23^ − 1. The number of sampling points per 1 bit was set to 10. The NRZ-OOK signal was loaded to an arbitrary waveform generator (Tektronix, Beaverton, USA, AWG 70002A) sampled at 100 MS/s. Therefore, the transmission rate of the NRZ-OOK signal was 10 Mb/s. The light from the white LED was modulated by the electrical NRZ-OOK signal using a bias-tee. Next, two kinds of light sources were used to generate noises from themselves. One was a fluorescent lamp (Philips Energy Saving CFL spiral lamp 12 W ES (E27)), and the other was a dimmable LED lamp (IKEA’s LEDARE GU10 LED bulb) with adjustable brightness. The optical power of the white LED modulated by the NRZ-OOK signal and the optical powers of the fluorescent lamp, and dimmable LED was all set equal to −5 dBm. A collimator (Edmund optics, LightPath 355561|15 mm Dia., 0.60 NA, BBAR (350–700 nm), molded aspheric lens) was used to uniformly transmit the lights generated from the three light sources to the avalanche photodiode (APD, Hamamatsu, Hamamatsu City, Japan, C12702-11, 100 MHz bandwidth). If the collimator is not used, the light from the light source will spread, and then the SNR of the received NRZ-OOK signal may be reduced. The important thing is that it is difficult to ascertain whether the amount of light emitted from the three different light sources is injected equally into the APD without a collimator. The optical wireless transmission distance was set to 1 m, and the received optical power was set to −10 dBm using an attenuator (Thorlabs, Newton, USA, LCC 1620) to prevent the optical saturation of the APD and then aligned to maximize the received optical power. The attenuator was used to measure the BER while varying the received optical power.

In an actual optical wireless environment, the transmission distance may be at least 1 m because it is highly likely that various types of light sources are installed on the ceiling. Therefore, the wireless transmission distance was set to 1 m in the experimental setup of Figure 2. For reference, since the optical power of the light source is inversely proportional to the square of the transmission distance, the optical SNR and the intensity of interference noise depend on the transmission distance.

The transmitted light was converted into an electric signal by optical to electrical (OE) conversion in APD, and then the waveform of the converted electric signal was captured at a real-time oscilloscope (Tektronix, Beaverton, USA, MSO 71604C) sampled at 250 MS/s. After this, the NRZ-OOK signal was recovered after reducing the noise generated from the ambient light source using the proposed ADE technique offline. At this time, the regularization parameter, λ mentioned in Equation (11), is set to 9. The four different signal waveforms presented at the bottom of the experimental setup show the waveforms measured at points Figure 2A–D, and two RF spectra (Figure 2E,F) under the four different signal waveforms show the noises generated at the fluorescent lamp and the dimmable LED at the optical power of −5 dBm. It can be seen that the recovered NRZ-OOK waveform (Figure 2D) after using the proposed technique was restored similarly to the output waveform (Figure 2B) from an arbitrary waveform generator (AWG). For reference, as shown in Figure 2, since the implemented optical wireless transmission link has a transmission rate of 10 Mb/s, the transmission rate is lower than that of other recent research results showing over 100 Mb/s. Here, our goal is not to maximize the transmission rate but to verify that the proposed technique can conceptually reduce the ambient light noise. Therefore, we simply implemented an optical wireless transmission link with a transmission rate of 10 Mb/s. In addition, Table 1 shows the experimental parameters and device-related information.

## 4. Experimental Results

**Figure 3:**
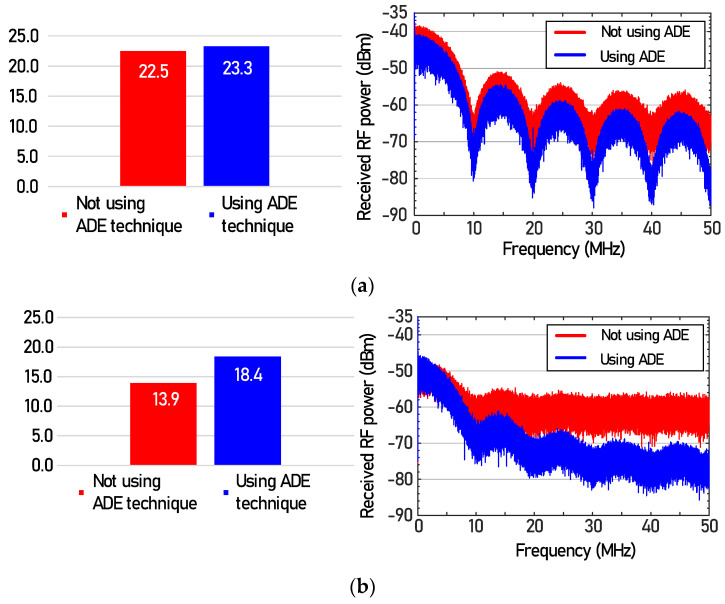
Signal−to−noise ratio (left) and RF spectrum (right) of NRZ−OOK signal before and after using ADE technique, (**a**) in case of transmitting only white LED light, (**b**) in case of transmitting fluorescent lamp, and dimmable LED as well as white LED light.

Figure 3 shows the signal-to-noise ratio (left) and RF spectrum (right) of the NRZ-OOK signal before and after using the proposed ADE technique. Figure 3a shows the SNR and RF spectrum of NRZ-OOK signal in case of transmitting only the white LED light modulated by the NRZ-OOK signal except for the fluorescent lamp and dimmable LED. Figure 3b shows the SNR and RF spectrum of the NRZ-OOK signal when the fluorescent lamp and dimmable LED light were transmitted. The part marked in red indicates the result where the proposed ADE method is not used, and the part marked in blue indicates the result of using the proposed ADE technique. The optical power of each light source was set to −5 dBm, and the received optical power at the APD was set to −10 dBm using an attenuator and collimator. As shown in Figure 3a, the SNR of the NRZ-OOK signal increased from 22.5 dB to 23.3 dB after using the ADE technique when only the white LED light is transmitted wirelessly to the APD. The improvement of SNR is because shot noise generated in APD due to the injection of white LED light was reduced by the ADE technique.

In Figure 3b, the SNR of 13.9 dB was observed when the ADE technique was not employed. We can see that the SNR of the NRZ-OOK signal was reduced by 8.5 dB than that of Figure 3a without using the ADE technique. This is because the interference noise (see the RF spectra at the bottom of Figure 2) and shot noise generated by the two light sources (fluorescent lamp and dimmable LED) were generated at APD. In addition, it was confirmed that the 4.5 dB SNR (from 13.9 dB to 18.4 dB) was improved with the help of the ADE technique.

**Figure 4:**
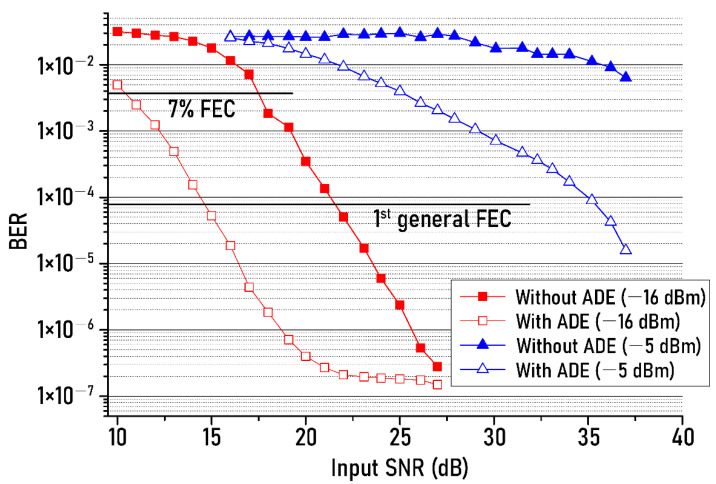
Bit error rate (BER) changes against the SNR of the input NRZ−OOK signal.

Figure 4 shows BERs of the recovered NRZ-OOK signal after optical wireless transmission, which was repeatedly measured while changing the SNR of the input NRZ-OOK signal. The blue line shows the measured BERs when the total optical power of the two ambient light sources (fluorescent lamp and dimmable LED) is −5 dBm, and the red line shows the measured BERs when the optical power of the two ambient light sources is −16 dBm. Filled red squares and filled blue triangles correspond to the BERs in case of not using the ADE technique, while open red squares and open blue triangles indicate the BERs of when the ADE technique is employed. When the optical power of the two light sources was −5 dBm (filled and open blue triangles), the BERs of the recovered NRZ-OOK signal were repeatedly measured while changing the SNR of the input NRZ-OOK signal from 10 dB to 27 dB. In addition, the BERs were measured while changing the SNR of input NRZ-OOK signal from 16 dB to 37 dB in case that the optical power of the two light sources was −16 dBm (filled and open red squares).

When the optical power of two light sources was −5 dBm, the SNR of the input NRZ-OOK signal should be at least 25 dB in order to transmit the modulated light of white LED successfully with the help of ADE and the 7% forward error correction (FEC) technique (threshold: 3.8 × 10^−3^ BER) at the same time. In addition, the SNR should be at least 35 dB if the proposed ADE is used with the first general FEC (threshold: 8.0 × 10^−5^ BER). At the two ambient lights (fluorescent lamp and dimmable LED) power of −16 dBm, the SNR gain of 7 dB was improved based on the 7% FEC threshold. In addition, it was found that the SNR gain has increased by 7.5 dB with the help of the first general FEC. For reference, in the case of a signal with a modulation index higher than OOK, the required SNR for successful transmission increases than NRZ-OOK. For example, to obtain the 1st general FEC threshold (3.8 × 10^−5^ BER), the pulse amplitude modulation (PAM)-4 signal requires about 7.5 dB more signal-to-noise ratio than the NRZ-OOK signal. Therefore, it is important to determine what kind of modulation format to use according to the wireless service to be operated in the system as well as the transmission environment.

**Figure 5:**
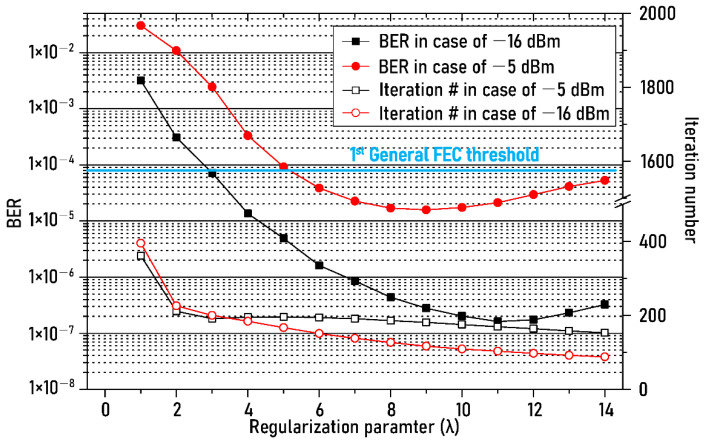
BER changes of recovered NRZ−OOK signal and iteration number against the regularization parameter.

On the other hand, since the regularization parameter mentioned in Equation (8) determines the shape of the recovered NRZ-OOK signal waveform, it is necessary to find out the impact of the regularization parameter on the transmission performance of the NRZ-OOK signal. Furthermore, as mentioned in Section 2.2, the complexity of DSP depends on the number of iterations calculation into the algorithm of the ADE technique. Hence, it is necessary for us to reduce the number of iterative calculations while maintaining a low BER. By the way, it is worth noting that the number of iterative calculation depends on the regularization parameter because it is executed until the output NRZ-OOK signal shows the waveform most similar to the input NRZ-OOK signal, and the difference between adjacent sampling points is less than the threshold determined in the algorithm. Therefore, we also checked how the number of iteration calculation would change by the change of the regularization parameter. Figure 5 shows the measured BERs (left y-axis) of recovered NRZ-OOK signal, and the number of iterative calculations (right y-axis) after ADE technique as the regularization parameter increases from 1 to 14. The received optical power of APD was fixed at −10 dBm. As shown in Figure 5, if the use of the FEC technique can be accomplished, in the case of the ambient light noise power of −16 dBm, the optical wireless transmission was achieved successfully within the regularization parameter range from 3 to 14 with the help of 1st generation FEC. In addition, it is possible to transmit the NRZ-OOK signal wirelessly within the regularization parameter range from 5 to 14 when the optical power of the ambient light was −5 dBm. In summary, the BER result in Figure 5 indicates that the proposed scheme is less dependent on the value of a specific regularization parameter.

In addition, the regularization parameters with minimum BER were measured as 9 and 11 at the optical power of each ambient light.

In terms of the number of iterative calculations, as the regularization parameter value increases to 14, the iterations number gradually decreases from 400 to 150 or 100 at the respective optical power of the ambient light. These results indicate that it is important to optimize the regularization parameter taking into account the number of iterative calculations in order to maximize transmission performance while reducing interference noise and shot noise using the proposed ADE technique in the real Li-Fi network.

In Equation (10) presented in Section 2, the Poisson distribution function was used as a weighting function to increase the reduction efficiency of shot noise. Therefore, it is necessary to find out whether the choice of the Poisson distribution function can help us to increase the reduction efficiency of shot noise.

**Figure 6:**
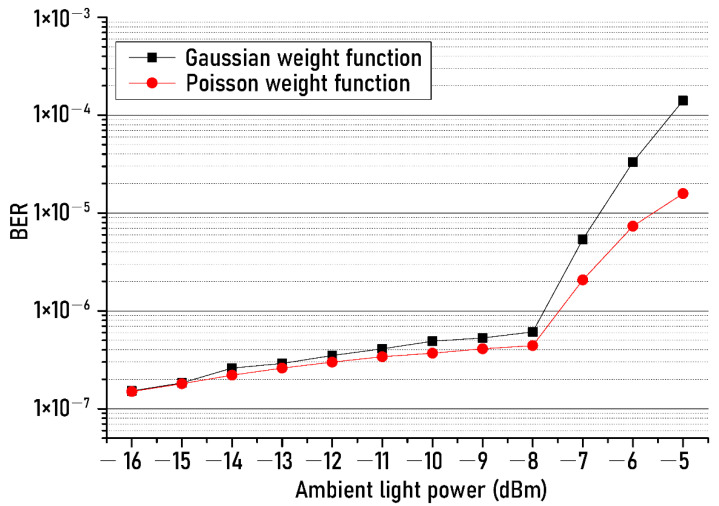
BER changes of recovered NRZ−OOK signal against two different weight functions.

Figure 6 shows the BER measured according to the ambient light power change for two different weighting functions. One of the weighting functions was a function with Gaussian distribution, and the other was a function showing Poisson distribution. As shown in Figure 6, it was observed that, within the full range of the ambient light power, the BERs when using the Poisson distribution function were less than the BERs in the case of using the Gaussian distribution function. In particular, the difference in BER between the two weighting functions gradually increased as the ambient light power exceeded −8 dBm. For example, at ambient light power of −5 dBm, the BER was reduced from 1.4 × 10^−4^ to 1.6 × 10^−5^ when the Poisson weight function was used. This result can be explained as follows: As the ambient light power increases, the shot noise having the Poisson characteristics increases. Therefore, the use of a weight function based on a Poisson distribution allows us to reduce shot noise more than when using a Gaussian distribution function.

**Figure 7:**
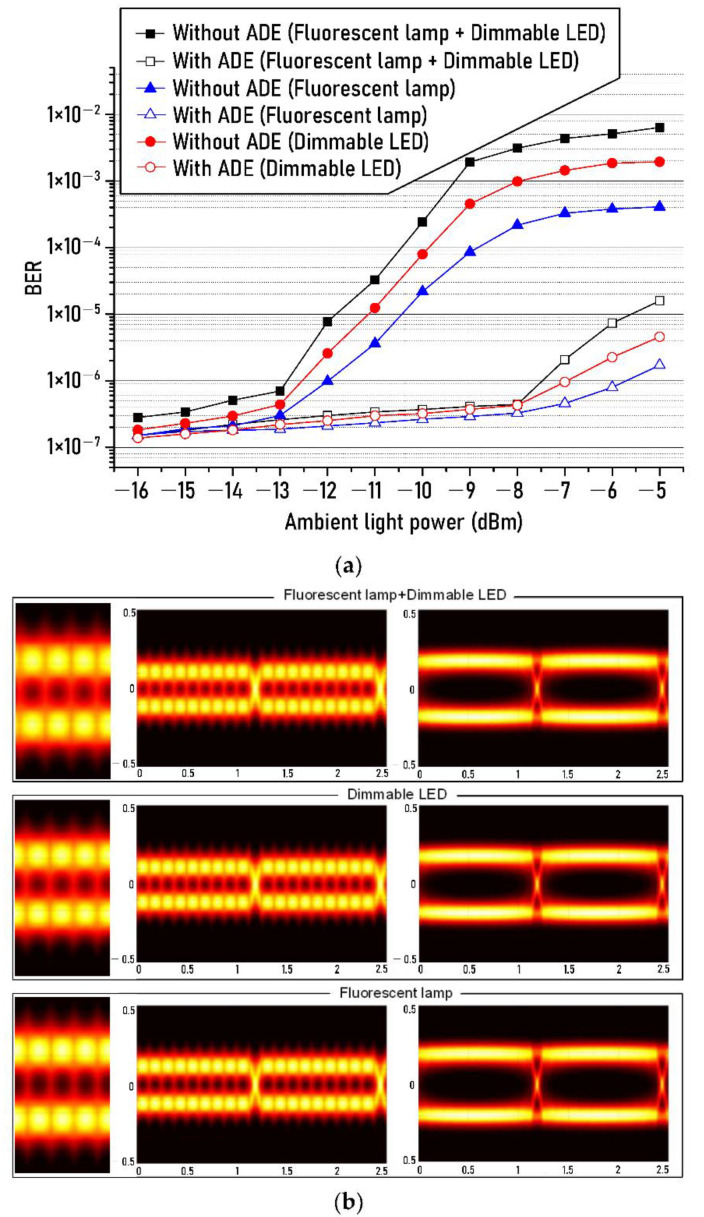
(**a**) BER changes of recovered NRZ−OOK signal as the optical power of ambient light increases from −16 dBm to −5 dBm, (**b**) eye diagrams at the ambient light power of −5 dBm, which were measured for three kinds of cases. Insets on the far left: enlarged eye patterns of each case before using the proposed ADE.

Figure 7 shows the measured BERs of recovered NRZ-OOK signal after the ADE technique as the optical power of ambient lights (fluorescent lamp and dimmable LED) increases from −16 dBm to −5 dBm. Eye diagrams present below the measured BER results were measured for three kinds of cases at −5 dBm ambient light power. The three cases here refer to: The first is when the white LED light and the lights of fluorescent lamp, and dimmable LED are transmitted simultaneously, the second is when only the white LED light and fluorescent lamp are transmitted, and the third is when only the white LED light, and dimmable LED light are transmitted. The received optical power after the optical wireless transmission was −10 dBm. The filled black squares and open black squares represent BER results of the first case before and after the ADE technique and filled blue triangles, and open blue triangles represent the BER results of the second case with and without the ADE technique. In addition, filled red circles and Open red circles indicate the BER results of the third case before and after the ADE technique.

As shown in Figure 7, it can be seen that the impact of the interference and shot noises generated from the dimmable LED on the transmission performance of the optical wireless link is greater than that of the fluorescent lamp within the entire range of the measured power of the ambient light. For example, when the optical power of the ambient light is −9 dBm, the BER in the case of the fluorescent lamp is 8.5 × 10^−5^, and under the impact of the dimmable LED, the BER is 4.5 × 10^−4^. This is because, as shown in the RF spectrum of interference noise generated from the ambient lights (fluorescent lamp and dimmable LED) presented at the bottom of the experimental setup in Figure 2, the number of the interference noise component of the dimmable LED was larger than that of the fluorescent lamp within the frequency range of 1 kHz. Therefore, the BER in the case of dimmable LED increases more than in the case of a fluorescent lamp due to the more generated interference noise components. Furthermore, for example, the optical power of the ambient light should be less than about −12 dBm in order to obtain a BER of 1 × 10^−5^ in case of not using the ADE technique. However, when the ADE technique is utilized, the optical power of the ambient light source should not exceed −6 dBm. These results tell us that even if the intensity of the ambient light source is increased by about 6 dB, the transmission performance can be accomplished sufficiently with the help of the ADE technique.

**Figure 8:**
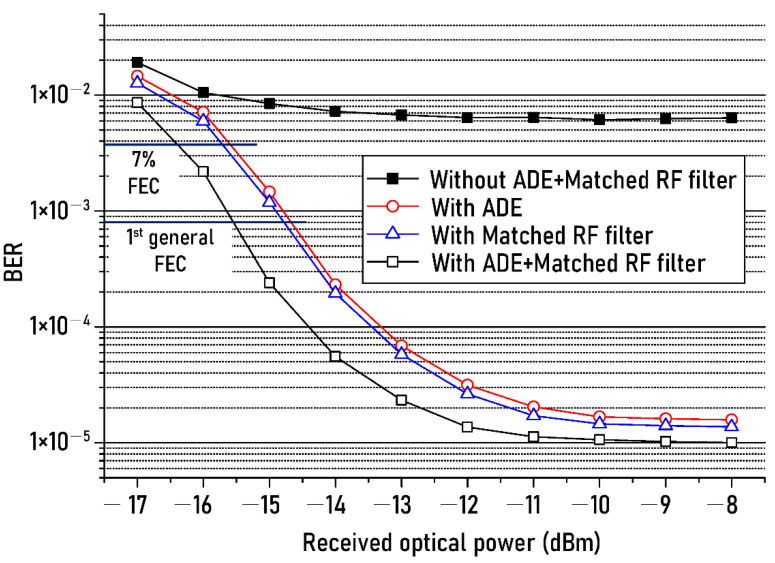
BER changes of recovered NRZ−OOK signal against the received optical power.

Figure 8 shows the measured BERs of the received NRZ-OOK signal as the received optical power increases from −17 dBm to −8 dBm. The performance of the matched filter was compared in order to objectively evaluate the transmission performance of the proposed ADE technique. Filled squares and open squares show the BERs change, respectively before and after using both the ADE technique and the matched RF filter, and the open circles show the BERs of when only the ADE technique is used, and the open triangles represent the BERs of when only the matched RF filter is employed.

As shown in Figure 8, the power penalty of less than 0.5 dB was observed due to the use of the ADE technique (open red circles) compared to the matched RF filter (open blue triangles). This result means that the transmission performance of the proposed ADE technique is hardly different from that of matched RF filter. In addition, it can be seen that when the simultaneous use of the ADE technique and the matched RF filter allows us to reduce the power penalty of 1 dB compared to the case of using only the ADE or only matched RF filter based on the 7% FEC threshold. The reduction of the power penalty of 1 dB was founded likewise in the case of the 1st general FEC threshold.

A typical method of filtering the baseband component is to use the matching filter presented in the result of Figure 8. Hence, far, it is known that the performance of improving the signal-to-noise ratio using a matching filter is the best. As shown in Figure 8, the proposed technique shows a power penalty of less than 0.5 dB compared to the method using a matching filter.

However, unlike the matching filter, since the proposed technique is based on DSP, there is no insertion loss caused by using the RF matching filter. In addition, since it is not necessary to know the header information of the input signal in advance to reduce interference noise, it can be used in various network systems using various protocols.

For reference, for data with a long PRBS length, the time to be affected by various noises generated during the transmission is longer than for data with a short pattern length. Therefore, the probability of generating errors in the detection process increases. This means that the BER increases as the pattern length increases. Accordingly, it is necessary to optimize between the PRBS length and the data rate.

In addition, the BER floor clearly occurred when the received optical power exceeded −10 dBm. This is due to the following reasons. As shown in Figure 4, the BERs of output NRZ-OOK signal were measured when the output power of the white-LED was set to −5 dBm and the received optical power was −10 dBm. In the case of using the proposed technique, the BER of 0.9 × 10^−5^ was measured at the input SNR of 37 dB. As reviewers know, the SNR of the input signal is constant regardless of the change in the received optical power because the output power of the white LED was fixed to −5 dBm. Therefore, as shown in Figure 8, even if the received optical power is increased to −10 dBm or more, the signal-to-noise ratio of the input NRZ-OOK signal does not change. Eventually, the BER does not decrease any more. In addition, to further reduce the BER, the following improvement methods can be considered. First, the optical SNR can be improved d by increasing the output optical power of the white LED over −5 dBm. Second, the BER can be reduced by improving the frequency response of the wireless optical transmission system by using a blue filter before receiving the transmitted optical signal or by additionally using an equalizer after optical detection.

## 5. Conclusions

We proposed and experimentally verified a technique to reduce interference noise and shot noise generated from ambient light sources in an optical wireless transmission system based on white LED. The proposed ADE technique takes advantage of the fact that the derivative change between adjacent sampling points of NRZ-OOK signal with a digital waveform is very different from them of interference noise and shot noise having a random and analog waveform. In addition, the proposed technique is implemented by adding a weight function that reflects the Poisson characteristic of shot noise. The main experimental results of the proposed ADE technique were as follows: the SNR of the input NRZ-OOK signal was improved by 7.5 dB based on the first generation FEC using the proposed ADE technique in the presence of the two ambient lights (fluorescent lamp and dimmable LED) with the optical power of −5 dBm. The BER of 1 × 10^−5^ was kept using the proposed ADE technique even when the optical power of the ambient light sources increased by 6 dB. The increase in power penalty less than 0.5 dB was observed compared to the matched RF filter. It was founded that the power penalty was more reduced by 1 dB by using the proposed ADE technique and the RF filter at the same time.

## Figures and Tables

**Table 1:** Experimental parameters and optical/electrical device.

Parameter	Value/Dimension	Unit
PRBS length	2^23^ − 1	-
Sampling points/bit	10	-
Sampling rate of AWG70002A	100	MS/s
Sampling rate of MSO 71604C	250	MS/s
Transmission rate	10	Mb/s
Wireless transmission length	1	Meter
Modulation format	NRZ-OOK	
Regularization parameter	9	-
Optical power of three optical source	−5	dBm
3 dB bandwidth of APD	100	MHz
Collimator	15 mm Dia., 0.60 NA, BBAR (350–700 nm)	-

## Data Availability

Not applicable.
